# Why I shrank my lab by half

**DOI:** 10.7554/eLife.82831

**Published:** 2022-09-05

**Authors:** Anne E Carpenter

**Affiliations:** 1 Broad Institute of MIT and Harvard Cambridge United States

**Keywords:** sparks of change, careers, principal investigator, health, research culture

## Abstract

A medical diagnosis sets a principal investigator on a new path.

Six months into 2020, I began having joint pain. It was mild but I knew that I should seek help just in case I was experiencing the first pangs of a serious disease. As physicians proposed potential diagnoses**,** I was suddenly confronted with the possibility that I might soon become unable to type or walk. My mind began swimming with thoughts about what this could imply for my future, and for my career.

My doctor finally settled on a likely diagnosis of psoriatic arthritis. No treatment was available unless the symptoms became significantly worse, but he said that one thing may help: reducing stress. I laughed at the suggestion. What a thing to say, during a pandemic, to a mother of five leading a 20-person research group! Still, I recognized that it was completely irrational to run my own health into the ground doing biomedical research to improve other people’s wellbeing. I knew that something had to change, but none of the obvious options were appealing.

Quitting entirely was an excruciating thought. I have been running a lab at the Broad Institute for over a decade, and I had just started to co-lead the NIH-funded Center for Open Bioimage Analysis. As one of the rare women in a computational field, I felt an obligation to stay. Above all, I find great joy in science. After years of feeling like I was keeping my head just barely above water, I was finally hitting my stride.

I considered reducing my hours, but I’d always been adamant about limiting my time in the lab. Even as a principal investigator, I typically put in 45–50 hours a week, apart from major deadlines. These boundaries already put pressure on my workday: reducing my hours without decreasing my workload would only create more stress.

Left with few options, I started thinking about scaling back my lab and reducing the number of scientists I employ and train. With fewer people to fund, I could cut down on the number of grant proposals and progress reports I had to write. It would also reduce how many projects I need to design and oversee to publication.

Still, most labs at my institution were already bigger than mine: if my lab became smaller, would people assume that I had grown complacent in mid-career, or that I couldn’t get grants? Would downsizing impact the quality and breadth of my research? And would a smaller lab survive inevitable fluctuations in funding? I spent six months thinking through options and discussing them with my leadership, which luckily was supportive and never showed a hint of disappointment. In the end, I decided to shrink my lab by half.

A former staff scientist, Beth Cimini, was just launching her own group: I transferred a major grant and half my personnel to her, as she had been instrumental in designing the research and mentoring lab members. With this move I will no longer work on the research which resulted in us creating CellProfiler, a tool that thousands of biologists now use to measure and analyse cell images; instead, I am dedicating myself to Cell Painting, a narrower but growing technology that has already yielded several clinical drug candidates.

In addition, I formally named a long-time staff scientist, Shantanu Singh, as co-leader of my now-smaller group; we are now officially the Carpenter—Singh lab! To me, this was an important step to recognize the leadership he had already been providing. We received so much support when we announced this decision on Twitter, with many wishing that more institutions would encourage the concept of a shared lab. There is no denying that it is an effective way to do science, especially at the interface of two fields like computational biology. And research is simply more fun with a senior colleague.

It has now been a year since the downsize. The workload has taken a while to slow down as papers from my former group are still being revised and published, but I’m starting to see signs of this new career phase. I’ve begun to catch up on reading literature, and I’m generally just less harried from day-to-day with deadlines. I’ve had to be careful to avoid filling in more work the moment there’s a bit of breathing room – a common pitfall for many academics – but for the first time since I launched my lab 15 years ago, I plan to use my full vacation time this year. Scientifically, the outcomes have been more positive than I could have imagined: it’s been incredibly satisfying to see Beth owning and growing her independent research vision, and to officially share the credit for the work of the new Carpenter—Singh lab with Shantanu. Best of all, I’ve yet to experience a relapse of symptoms.

The world of science involves a lot of comparison, and the size of one’s lab is often used as a marker of success. I hope that even in the absence of a medical crisis, others will have the confidence and support to re-evaluate how to reduce stress in their environment and measure scientific success in a more meaningful and personal way.

## Share your experiences

This article is a Sparks of Change column, where people around the world share moments that illustrate how research culture is or should be changing. Have an interesting story to tell? See what we’re looking for and the best ways to get in touch here.

